# Supplementary figures and images for: Sicegar: R package for sigmoidal and double-sigmoidal curve fitting
Source: PeerJ. 2018 Jan 16;6:e4251. doi: 10.7717/peerj.4251 (PMC5774301; doi:10.7717/peerj.4251)

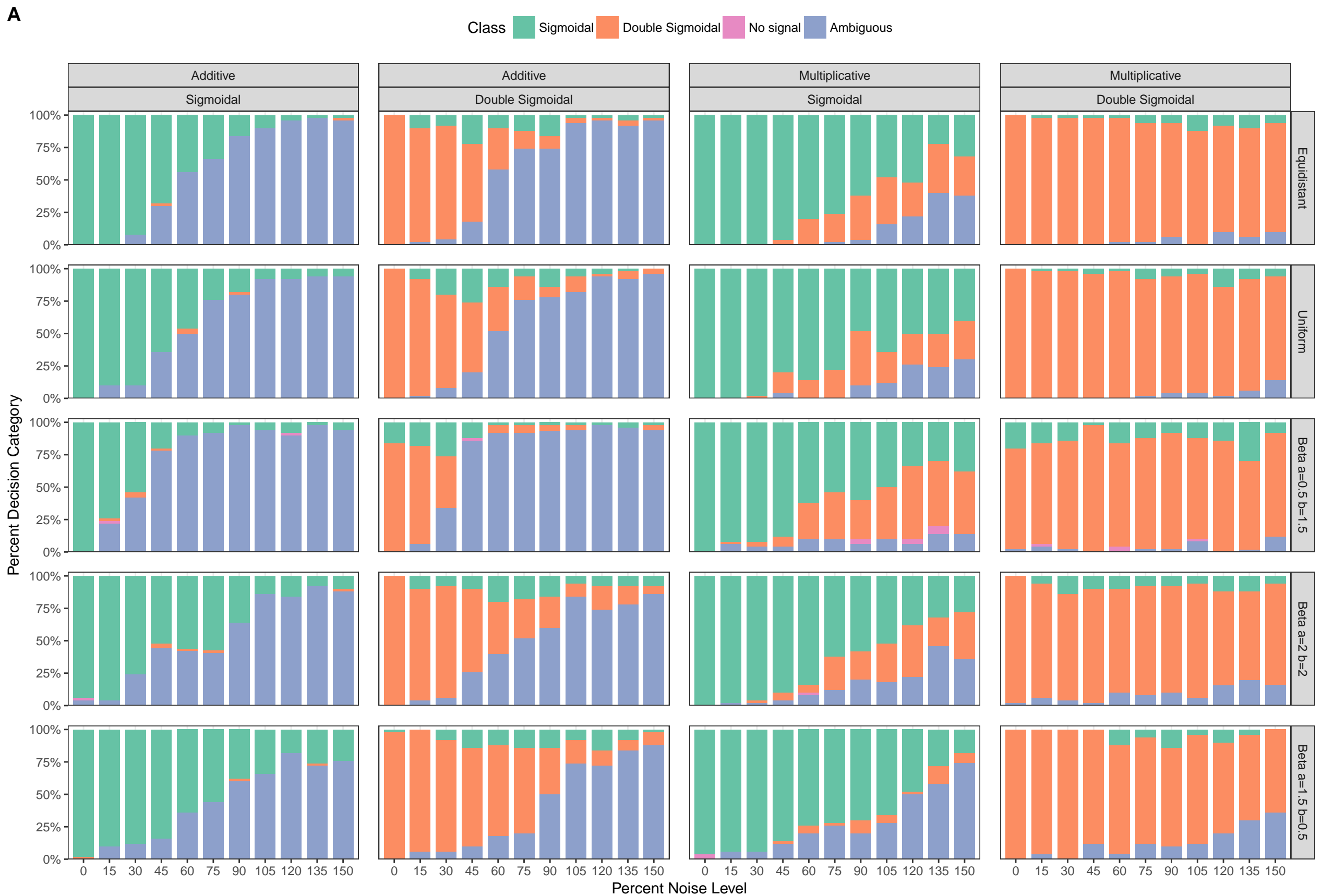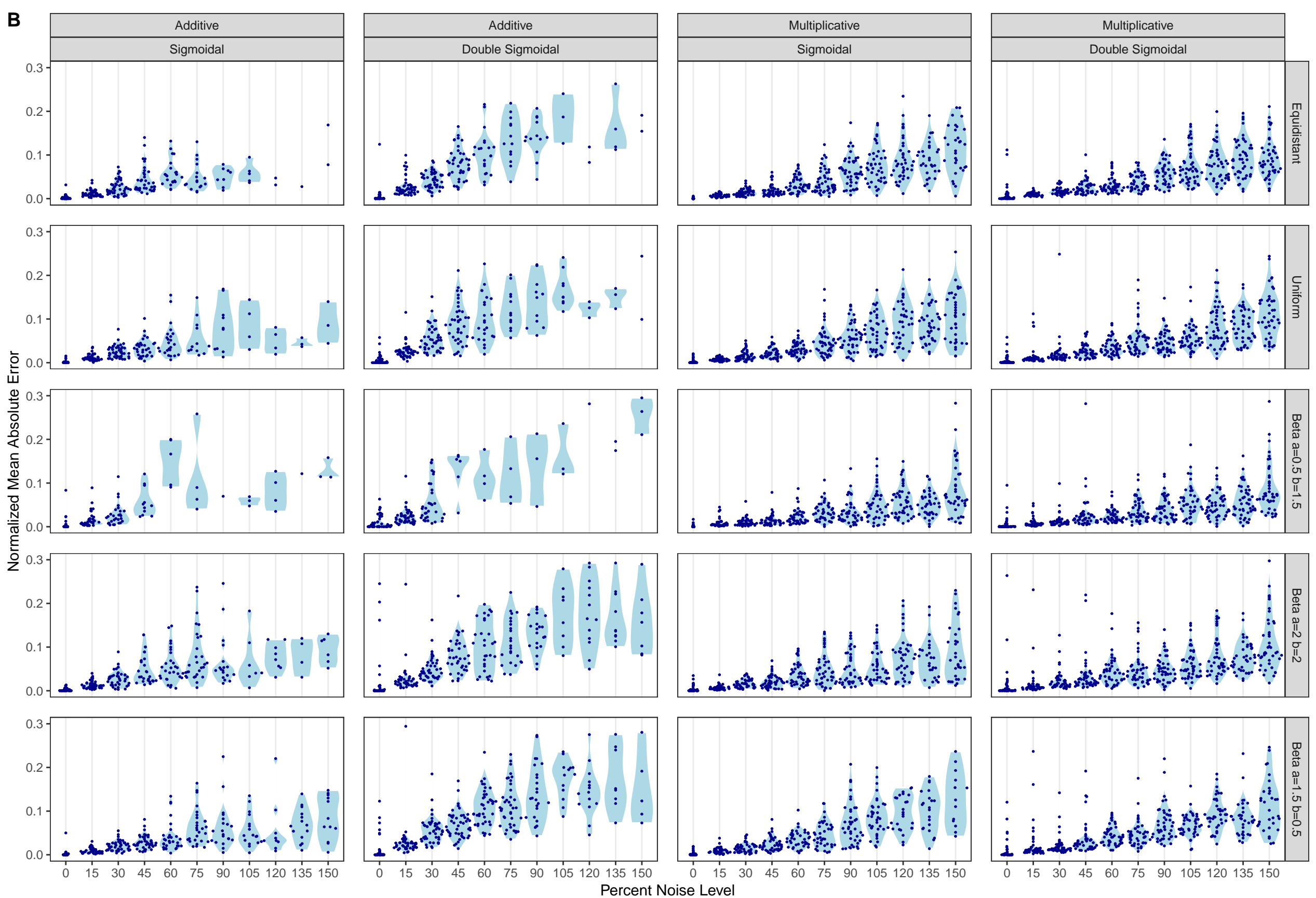

Supplement: Supplemental Information 1 — Noise levels are measured relative to the maximum intensity of the simulated curves. The temporal sampling regimes are: (i) equidistant sampling, (ii) random sampling from a uniform distribution, (iii)–(v) random sampling from beta distributions creating higher sampling densities at the beginning, center, and end of the time course. The parameter sets for the beta distributions are {alpha = 0.5, beta = 1.5} (mode at the beginning), {alpha = 2, beta = 2} (mode at the center), and {alpha = 1.5, beta = 0.5} (mode at the end). (A) sicegar tends to either recover the original type of curve correctly (for small to moderate amounts of noise) or label the dataset as ambiguous (at high noise levels). Performance is worse when sampling is concentrated towards the center or end of the time course (bottom two rows). (B) The difference between the original data (without noise) and the fitted curve is near zero for low noise levels and increases gradually and slowly for higher noise levels. [file peerj-06-4251-s001.pdf]
